# Supplementary figures and images for: Best in the company of nearby males: female success in the threatened cycad, Zamia portoricensis
Source: PeerJ. 2018 Jul 24;6:e5252. doi: 10.7717/peerj.5252 (PMC6063211; doi:10.7717/peerj.5252)

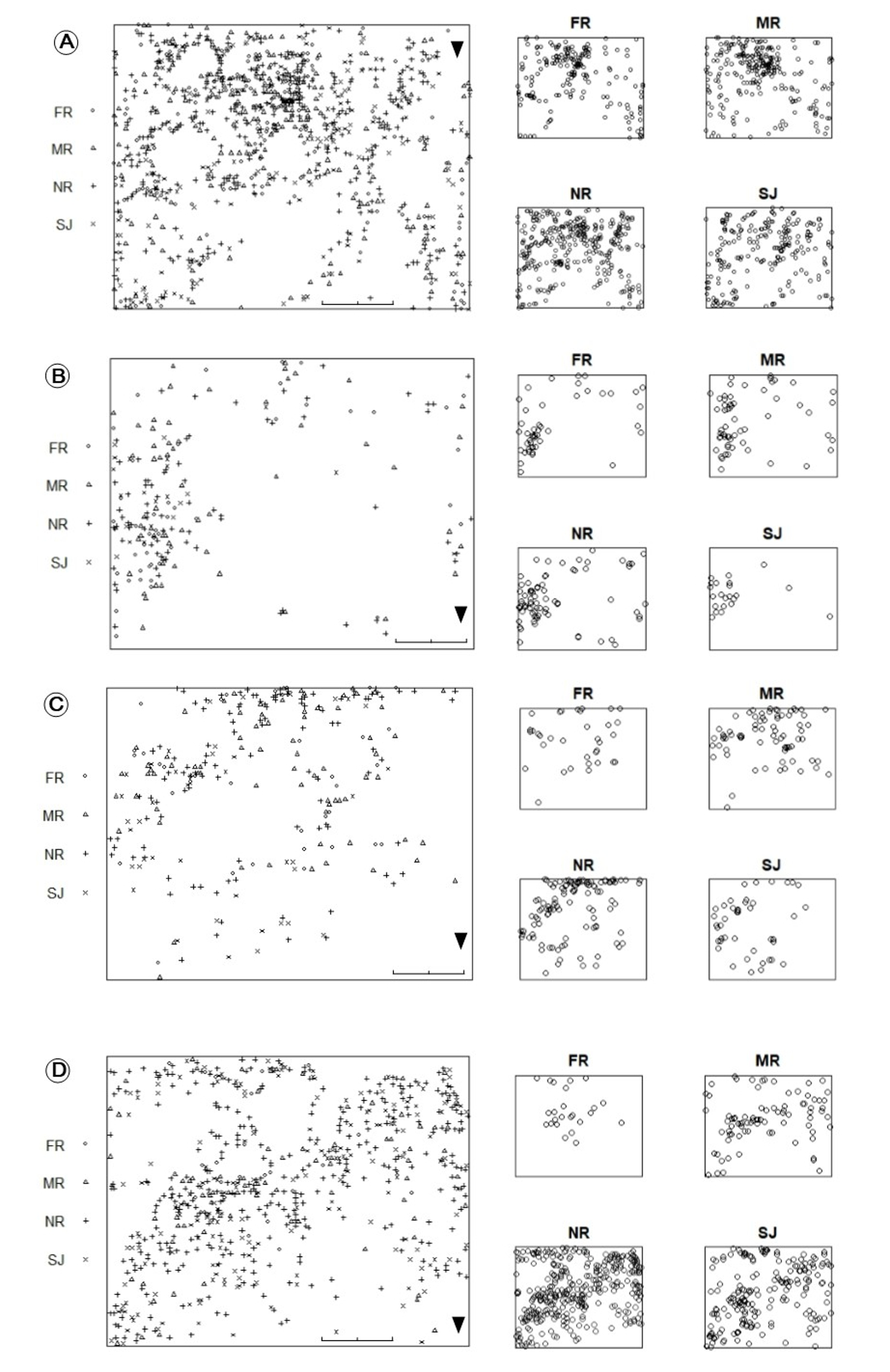

Supplement: Figure S1 — (A) El Tamarindo plot ET1. (B) El Tamarindo plot ET2. (C) Susúa State Forest plot SF1. (D) Susúa State Forest plot SF2. FR, female reproductive plants; MR, male reproductive plants; NR, non-reproductive plants; SJ, seedlings and juveniles. [file peerj-06-5252-s006.png]
