# Supplementary material for: Best in the company of nearby males: female success in the threatened cycad, Zamia portoricensis
Source: PeerJ. 2018 Jul 24;6:e5252. doi: 10.7717/peerj.5252 (PMC6063211; doi:10.7717/peerj.5252)
Supplement: Table S1 — Size was measured as the number of leaflets in the longest leaf. ET, El Tamarindo population plots; SF, Susúa State Forest plots; SE, standard error. [file peerj-06-5252-s007.docx]

Size distribution of males and females of *Zamia portoricensis* at El Tamarindo and Susúa State Forest sites. Size was measured as the number of leaflets in the longest leaf. ET = El Tamarindo population plots; SF = Susúa State Forest plots; SE = standard error.

|  | ET1 | | ET2 | | SF1 | | SF2 | |
| --- | --- | --- | --- | --- | --- | --- | --- | --- |
| Size class | Male | Female | Male | Female | Male | Female | Male | Female |
| 10-20 | 9 | 0 | 13 | 1 | 3 | 0 | 5 | 0 |
| 21-30 | 72 | 12 | 13 | 7 | 8 | 0 | 27 | 0 |
| 31-40 | 102 | 58 | 21 | 9 | 32 | 10 | 34 | 2 |
| 41-50 | 57 | 81 | 10 | 16 | 22 | 14 | 11 | 14 |
| 51-60 | 9 | 27 | 2 | 12 | 7 | 10 | 5 | 6 |
| 61-70 | 1 | 7 | 0 | 1 | 1 | 0 | 1 | 0 |
| > 70 | 0 | 2 | 0 | 0 | 0 | 0 | 0 | 2 |
| Total | 250 | 187 | 59 | 46 | 73 | 34 | 83 | 24 |
| Mean size | 35.33 | 43.33 | 31.47 | 42.41 | 38.89 | 45.29 | 34.10 | 49.5 |
| SE | 8.76 | 9.13 | 10.23 | 11.13 | 9.81 | 7.57 | 9.81 | 10.77 |
| % 10 - 20 | 3.6 | 0 | 22.03 | 2.17 | 4.11 | 0 | 6.02 | 0 |
| % 21 - 30 | 28.88 | 6.42 | 22.03 | 15.22 | 10.96 | 0 | 32.53 | 0 |
